# NOD1/NOD2 and RIP2 Regulate Endoplasmic Reticulum Stress-Induced Inflammation during *Chlamydia* Infection

**DOI:** 10.1128/mBio.00979-20

**Published:** 2020-06-02

**Authors:** Oanh H. Pham, Bokyung Lee, Jasmine Labuda, A. Marijke Keestra-Gounder, Mariana X. Byndloss, Renée M. Tsolis, Stephen J. McSorley

**Affiliations:** a Center for Immunology and Infectious Diseases, Department of Anatomy, Physiology and Cell Biology, School of Veterinary Medicine, University of California Davis, Davis, California, USA; b Department of Medical Microbiology and Immunology, School of Medicine, University of California at Davis, Davis, California, USA; Cornell University

**Keywords:** *Chlamydia*, innate immunity, NOD, ER stress, inflammation

## Abstract

The inflammatory response to *Chlamydia* infection is likely to be multifactorial and involve a variety of ligand-dependent and -independent recognition pathways. We previously reported the presence of NOD1/NOD2-dependent endoplasmic reticulum (ER) stress-induced inflammation during Chlamydia muridarum infection *in vitro*, but the relevance of this finding to an *in vivo* context is unclear. Here, we examined the ER stress response to *in vivo Chlamydia* infection. The induction of interleukin 6 (IL-6) production after systemic *Chlamydia* infection correlated with expression of ER stress response genes. Furthermore, when tauroursodeoxycholate (TUDCA) was used to inhibit the ER stress response, an increased bacterial burden was detected, suggesting that ER stress-driven inflammation can contribute to systemic bacterial clearance. Mice lacking both NOD1 and NOD2 or RIP2 exhibited slightly higher systemic bacterial burdens after infection with *Chlamydia*. Overall, these data suggest a model where RIP2 and NOD1/NOD2 proteins link ER stress responses with the induction of *Chlamydia*-specific inflammatory responses.

## Abstract (precis)

Understanding the initiation of the inflammatory response during *Chlamydia* infection is of public health importance given the impact of this disease on young women in the United States. Many young women are chronically infected with *Chlamydia* but are asymptomatic and therefore do not seek treatment, leaving them at risk of long-term reproductive harm due to inflammation in response to infection. Our manuscript explores the role of the endoplasmic reticulum stress response pathway initiated by an innate receptor in the development of this inflammation.

## INTRODUCTION

Chlamydia trachomatis is an obligate intracellular bacterium that causes a sexually transmitted disease that is increasingly common in the United States (1). More than 1.7 million *Chlamydia* infections were reported in 2017, representing a 6.9% increase over 2016 statistics and the largest number of annual infections ever reported to the U.S. Centers for Disease Control (CDC) for a single condition (1). Most *Chlamydia* infections are initially asymptomatic and thus go untreated, allowing the development of pelvic inflammatory disease (PID) in a proportion of infected women (2). This subclinical disease can eventually cause pelvic pain and long-term reproductive complications for infected individuals. Given the high number of infections in otherwise healthy young women and the potential for serious reproductive pathology, it is vital to achieve a detailed mechanistic understanding of *Chlamydia*-induced inflammation (3–5).

The inflammatory response to *Chlamydia* infection is initiated by host sensing of replicating bacteria in infected tissues (4). Studies in the mouse model point to an important role for inflammatory cytokines in the genesis of reproductive tract pathology (6–9). Indeed, whole exosome sequencing in women with PID identified several genes in the interleukin 1 (IL-1) signaling pathway associated with infertility (10). Excessive production of type I interferons and tumor necrosis factor alpha (TNF-α) also increases reproductive tract pathology in mice (11, 12). The most likely source of these inflammatory mediators is the local tissue macrophage and neutrophil response to *Chlamydia* infection of the epithelial layer (7, 13). However, cell-autonomous responses by infected epithelial cells are also likely to make a substantial contribution to the inflammatory environment (4). There is a wide variety of microbial sensors that could potentially drive cytokine release and could therefore be responsible for initiation of this pathology. It is important to develop a detailed understanding of which of these pathways is activated and contributes to reproductive tract pathology in infected women.

Toll-like receptors are an evolutionary conserved family of receptors that recognize microbe-derived and certain host ligands to initiate an inflammatory response (14). Toll-like receptor 2 (TLR2) has been identified as an important receptor involved in the induction of IL-6, IL-8, granulocyte-macrophage colony-stimulating factor (GM-CSF), and TNF-α by epithelial cells or macrophages in response to *Chlamydia* infection (15, 16). As might be expected, TLR2-deficient mice displayed reduced reproductive tract pathology compared to that of wild-type mice (15), confirming this sensor as a critical component of inflammatory responses. In contrast to a pathogenic role for TLR2 signaling, TLR3-deficient mice demonstrate enhanced bacterial shedding and hydrosalpinx development, suggesting that TLR3 promotes a host-protective response and bacterial clearance (17). The cytosolic sensor STING has also been shown to respond to *Chlamydia* infection, causing type I interferon production via recognition of double-stranded DNA and cyclic di-AMP (18, 19). Another group of cytosolic pattern recognition receptors are the NOD-containing proteins, NOD1 and NOD2. Both of these NOD molecules have been reported to induce cytokine secretion during *Chlamydia* infection (18, 20), but the ligands responsible have yet to be clearly delineated. Although NOD1-deficient mice displayed similar infection rates and reproductive tract pathology to those of wild-type mice (21), both NOD1- and NOD2-deficient mice display deficient clearance of Chlamydia pneumoniae (22), suggesting that these sensors are likely to play some role in the inflammatory response during *Chlamydia* infection.

The cytosolic receptors NOD1 and NOD2 can respond to bacterial peptidoglycan and activate NF-κB using RIP2, leading to the production of multiple inflammatory mediators (23). While NOD 1 and NOD2 can respond to bacterial peptidoglycan, these sensors can also detect cytoskeletal modifications initiated by a variety of intracellular bacteria (24). Interestingly, NOD1 and NOD2 are also involved in the induction of inflammatory responses to viruses and parasites that lack the known ligands that could initiate activation pathways for NOD-mediated inflammatory responses. *Chlamydia* have long been known to replicate within an intracellular compartment closely associated with the host endoplasmic reticulum (ER), where the bacteria gain access to host lipids and other metabolites (25). One consequence of this ER interaction is the potential to induce the unfolded protein response (UPR), a host response that seeks to reduce translation and initiate ER repair. Induction of the UPR is associated with the initiation of an inflammatory response that was recently shown to require NOD1/2 sensing (26). Thus, NOD1/2 might initiate ligand-independent induction of UPR-induced inflammation as a consequence of an ER stress response induced by *Chlamydia* infection (27). Indeed, we have previously reported NOD1/NOD2-dependent ER stress-induced inflammation during Chlamydia muridarum infection *in vitro* (26).

Here, we examined the ER stress response in the context of *Chlamydia* infection *in vivo*. Chlamydia muridarum induction of IL-6 production correlated with the induction of ER stress response genes. When tauroursodeoxycholate (TUDCA) was used to inhibit this ER stress response, an increased bacterial burden was detected in *Chlamydia*-infected mice, suggesting that ER stress-driven inflammation contributes to bacterial clearance. Mice lacking both NOD1/NOD2 or RIP2, an adaptor protein of NOD1/NOD2, exhibited higher bacterial burdens after infection with *Chlamydia* and an NOD1/2-dependent TUDCA-sensitive inflammatory response. Overall, these data suggest that RIP2 and NOD1/NOD2 proteins serve to link ER stress induced by *Chlamydia* infection with the induction of inflammatory responses.

## RESULTS

### Chlamydia infection induces the unfolded protein response and IL-6 production.

**FIG 1:**
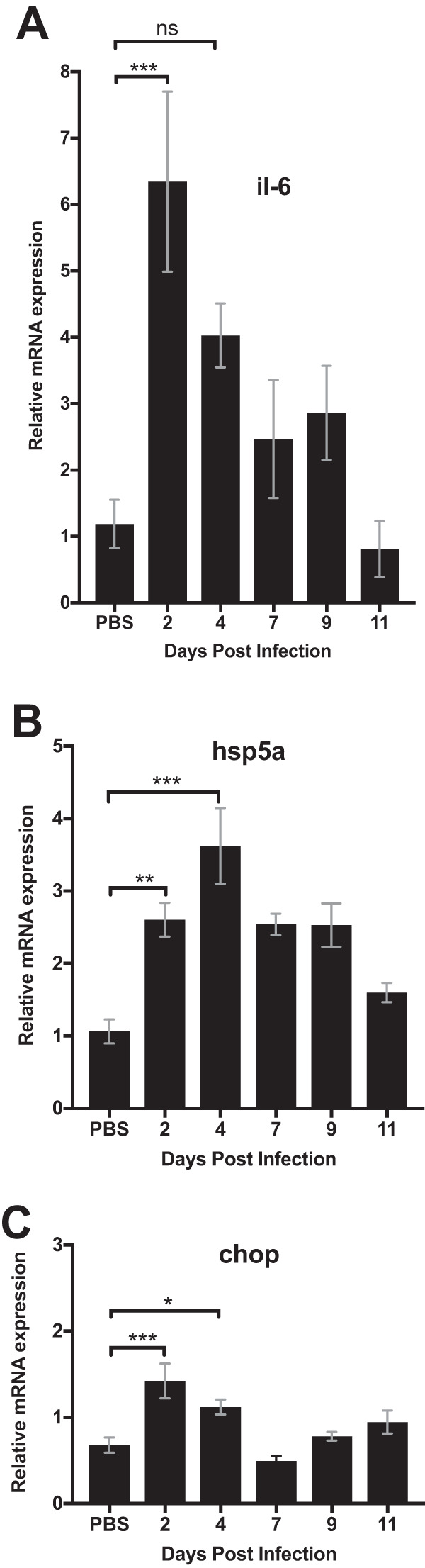
C. muridarum infection induced IL-6 and ER stress gene expression. C57BL/6 mice were infected intravenously (i.v.) with 2 × 10^5^ Chlamydia muridarum. Spleens were collected at different time points after the infection to determine mRNA levels of *il-6* (A), *chop* (B), and *hsp5a* (C) by quantitative PCR. There were at least three mice per group. Data shown are representative of two experiments. Statistical significance was determined by one-way analysis of variance (ANOVA) with a Holm-Sidak’s multiple-comparison test. Error bars represent the means ± standard errors of the means (SEMs). *, *P* < 0.05; **, *P* < 0.01; ***, *P* < 0.001; ns, not significant.

We previously reported that infection of HeLa cells with C. muridarum induces *il-6* mRNA expression that was blunted by treatment with the interferon gene regulatory element 1 alpha (IRE1α) kinase inhibitor KIRA6, or a dominant negative form of RIP2 (26). This *in vitro* data suggested that NOD1/NOD2/RIP2 signaling detects a host ER stress response to *Chlamydia* infection and induces inflammation. To extend these data to an *in vivo* model, we examined the induction of *il-6* and two genes that are regulated by ER stress sensors, *hsp5a* and *chop*. As expected, systemic infection of C57BL/6 mice with C. muridarum caused an elevation of *il-6* mRNA in the spleen within 2 days of infection (Fig. 1A). Simultaneously, C. muridarum infection also induced the UPR, as indicated by increased detection of *hsp5a* transcripts in the spleen (Fig. 1B), a gene regulated by ER stress sensors IRE1α and ATF6 (26). A transient increase in the expression of *chop* was also detected, 2 days after infection; however, this was not sustained (Fig. 1C). The expression of *chop* is controlled by a different ER stress sensor, PERK (26). Thus, the induction of *il-6* mRNA by C. muridarum infection correlates with the induction of UPR-responsive genes, particularly, *hsp5a*.

### Inhibition of ER stress in *Chlamydia*-infected mice reduces IL-6 production.

**FIG 2:**
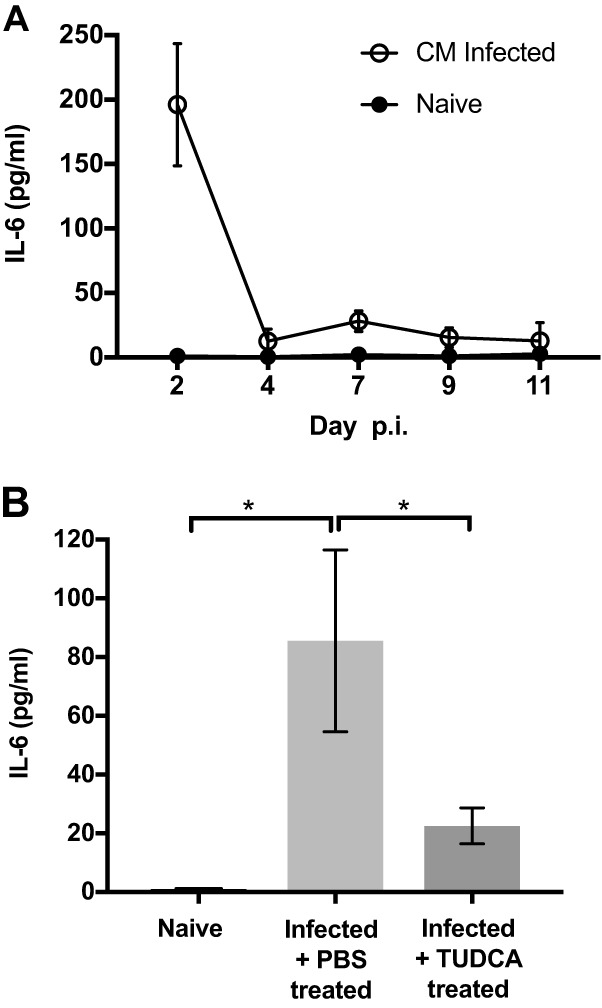
Inhibition of ER stress by TUDCA results in reduced IL-6 concentration in serum. C57BL/6 mice were infected intravenously (i.v.) with 2 × 10^5^ Chlamydia muridarum. (A) Spleen and serum were collected at different time points after the infection to determine the concentration of circulating IL-6. (B) Infected mice were administered i.p. with TUDCA at day 1 postinfection. Mice were euthanized at day 2 postinfection(p.i.). Sera were collected to determine the concentration of IL-6. Data shown represent the result from two separate experiments containing at least 3 mice per group. Statistical significance was determined by one-way ANOVA with a Holm-Sidak’s multiple-comparison test. Error bars represent the means ± SEMs. *, *P* < 0.05.

Given the correlation between the ER stress response and IL-6 production following *Chlamydia* infection, we examined whether these events might be connected. Although *il-6* mRNA remained elevated for several days in the spleen after infection (Fig. 1A), elevated serum IL-6 protein was primarily detected at day 2 postinfection (Fig. 2A). Using this convenient time point, we examined whether the ER stress inhibitor TUDCA affected the production of IL-6 in response to *Chlamydia* infection. Again, IL-6 was consistently elevated 2 days after *Chlamydia* infection, but this response was reduced significantly in *Chlamydia*-infected mice treated with TUDCA (Fig. 2B). In summary, these data suggest that the *in vivo* production of IL-6 in response to *Chlamydia* infection requires the induction of an ER stress response.

### RIP2 is required for proinflammatory response in C. muridarum infection.

**FIG 3:**
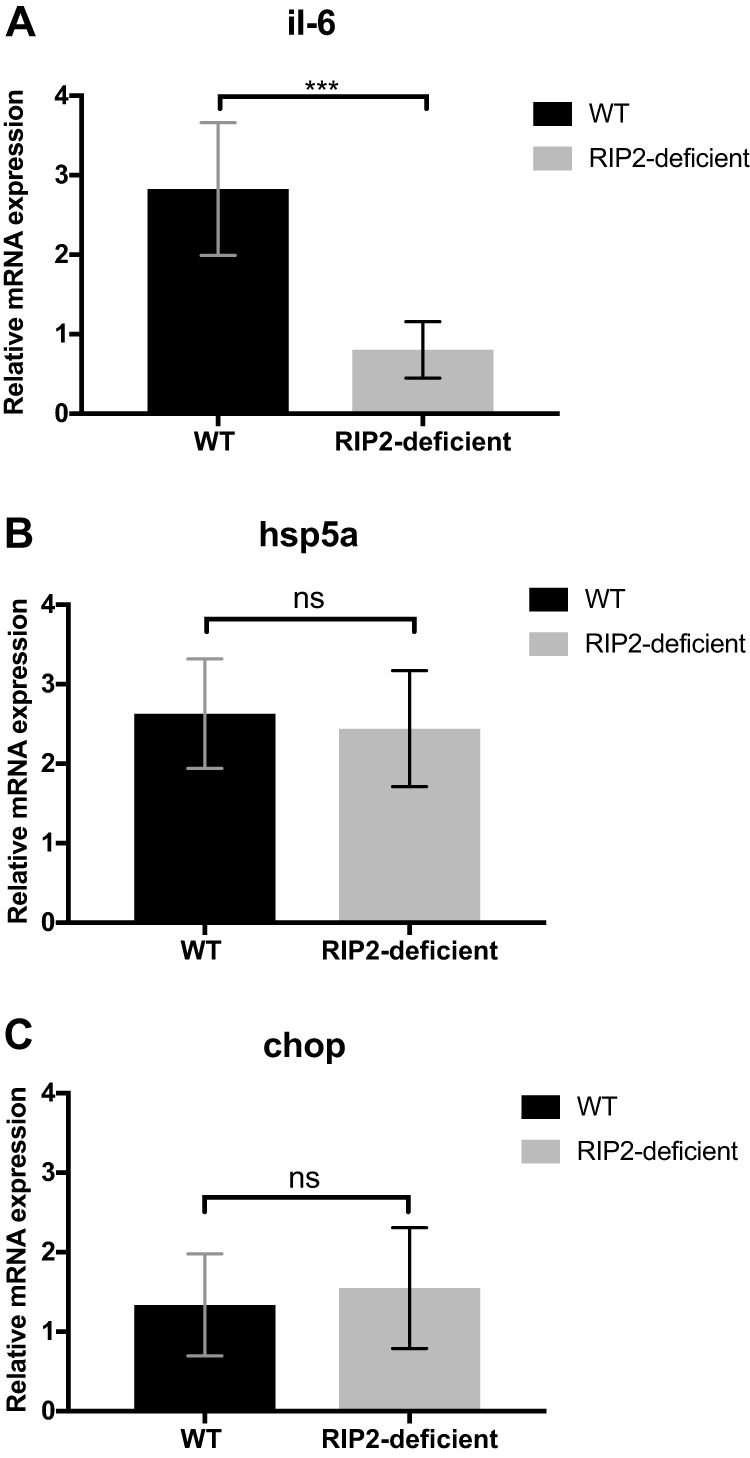
RIP2 depletion reduced IL-6 but not ER stress gene expression. C57BL/6 and RIP2^−/−^ mice were infected intravenously (i.v.) with 2 × 10^5^ Chlamydia muridarum. Infected mice were administered TUDCA or phosphate-buffered saline (PBS) as a control i.p. daily from day 1. At day 7 postinfection, spleens were collected to determine the level of *il-6* (A), *chop* (B), and *hsp5a* mRNA (C) by quantitative PCR. Data shown were combined from two experiments containing at least 3 mice per group. Statistical analyses were performed by Student's *t* test. Significance testing was performed with the nonparametric Mann-Whitney test. ***P < 0.01; ns, not significant. Error bars represent the means ± SEMs.

We recently documented a signaling pathway that links ER stress sensing and inflammation (26). This pathway required NOD1/NOD2 signaling and the adaptor protein RIP2 downstream of the sensor IRE1α (26). Thus, we examined whether IL-6 production in response to *Chlamydia* infection required the expression of RIP2. Indeed, levels of *il-6* mRNA in spleens of *Chlamydia*-infected RIP2^−/−^ mice were lower than in *Chlamydia*-infected wild-type (WT) mice (Fig. 3A). As expected, the absence of RIP2 did not affect transcript levels of ER stress genes *hsp5a* (Fig. 3B) or *chop* (Fig. 3C), since neither of these products of the UPR are dependent on RIP2. These data support the important role for RIP2 for the activation of proinflammatory responses during C. muridarum infection *in vivo*.

### Inhibition of ER stress or absence of RIP2 allows higher bacterial burdens *in vivo*.

**FIG 4:**
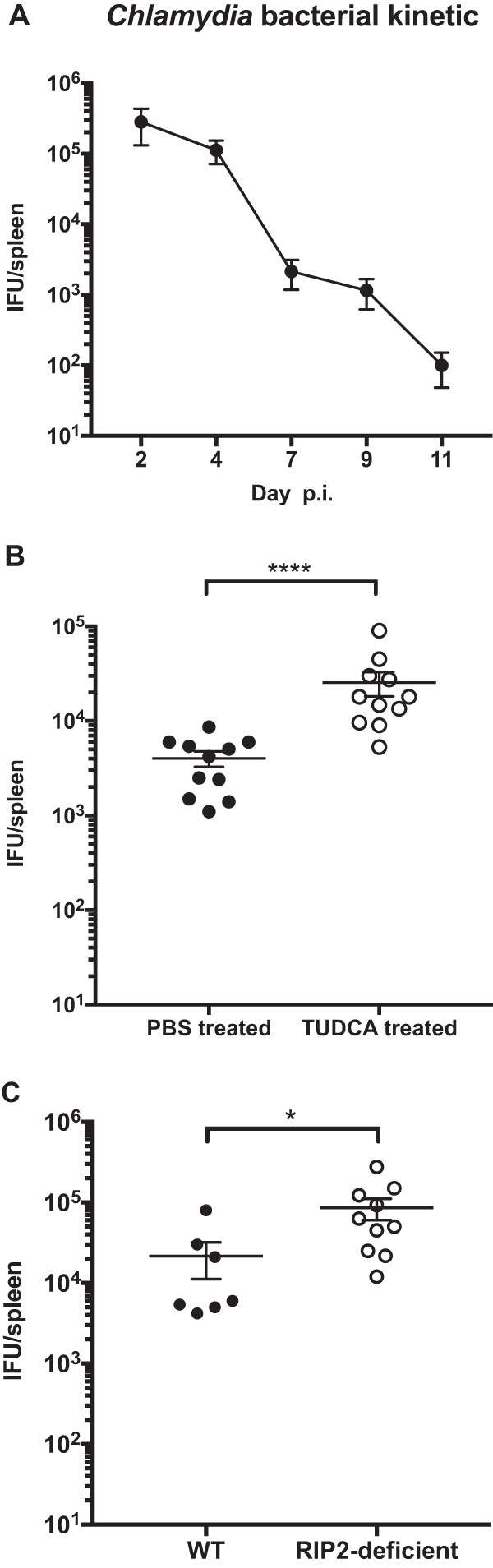
Inhibition of ER stress resulted in higher bacterial burden. C57BL/6 mice were infected intravenously (i.v.) with 2 × 10^5^ Chlamydia muridarum. (A) At various time points postinfection, inclusion-forming units (IFU) were enumerated by plating supernatants from tissue homogenates on HeLa 229 cells. (B) *Chlamydia*-infected mice were administered TUDCA or PBS as a control i.p. daily from days 1 to 6 p.i. At day 7 postinfection, mice were euthanized, and spleens were collected to determine bacterial burden. (C) C57BL/6 and RIP2^−/−^ mice were infected intravenously (i.v.) with 2 × 10^5^ Chlamydia muridarum. At day 7 postinfection, mice were euthanized, and spleens were collected to determine bacterial burden. Data shown are combined from two experiments containing at least 3 mice per group. Statistical analyses were performed by Student's *t* tests. Significance testing was performed with the nonparametric Mann-Whitney test. *, *P* < 0.05; ****, *P* < 0.0001. Error bars represent the means ± SEMs.

Given this link between the ER stress response and RIP2 for *il-6* mRNA associated with *Chlamydia* infection, it was of interest to examine whether this pathway affected bacterial clearance. To select an appropriate time point, we initially examined the kinetics of bacterial clearance from the spleen after systemic C. muridarum infection. Bacterial numbers were highest around 2 days postinfection but remained detectable at day 7 (Fig. 4A). Using this later time point, we compared bacterial burdens in wild-type mice administered TUDCA and also in RIP2-deficient mice. Consistent with the observed effect of TUDCA on IL-6 production, *Chlamydia*-infected C57BL/6 mice treated with TUDCA exhibited significantly higher bacterial burdens than infected C57BL/6 mice (Fig. 4B). Similarly, *Chlamydia*-infected RIP2-deficient mice had higher bacterial burdens in the spleen than WT C57BL/6 mice (Fig. 4C). Thus, the ER stress response and the RIP2-dependent pathway are essential components for optimal clearance of C. muridarum.

### NOD1/NOD2 depletion affected bacterial clearance in early infection.

**FIG 5:**
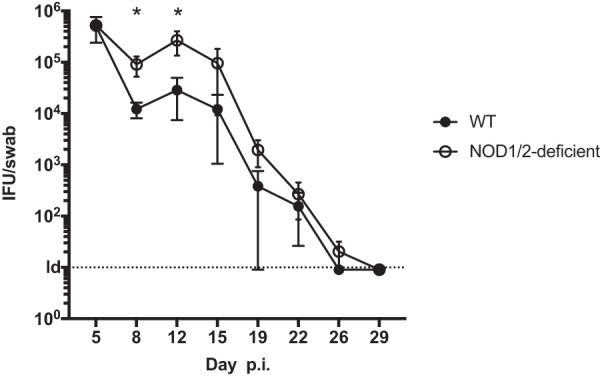
NOD1/NOD2 depletion hindered bacterial clearance at early *Chlamydia* infection. C57BL/6 mice and NOD1^−/−^ NOD2^−/−^ mice were infected intravaginally with 10^5^ C. muridarum/mouse. At various time points postinfection, bacterial burden in the lower genital tract was measured by vaginal swabs. Statistical analyses were performed by Student's *t* tests. Significance testing was performed with the nonparametric Mann-Whitney test. *, *P* < 0.05. Error bars represent the means ± SEMs.

Since RIP2 is an adaptor protein downstream of NOD1 and NOD2, it was of interest to examine the effect of combined NOD1 and NOD2 deficiency on *Chlamydia* clearance. Wild-type and NOD1/NOD2-deficient mice were infected intravaginally with C. muridarum, and bacterial shedding was determined by swabbing at multiple time points over the course of infection. NOD1/NOD2-deficient mice displayed a modest impairment of bacterial clearance, as evident by higher bacterial shedding during the early stage of infection (Fig. 5). At later time points, this modest impairment was overcome (Fig. 5). Thus, NOD1/2 signaling contributes to optimal resolution of *Chlamydia* infection, but there are likely to be other innate sensors that compensate for the absence of this response to effect final clearance from the host.

## DISCUSSION

There are numerous membrane-associated and cytosolic sensors that determine how an infected host detects different classes of microbial pathogens. These responses most likely work cooperatively and redundantly to induce a robust inflammatory response that leads to pathogen control. In the case of *Chlamydia*, these innate sensing pathways play a major role in the initiation of immune pathology associated with reproductive tract infection (3–5). Our data add to this knowledge by demonstrating a role for NOD receptors as sensors of the ER stress response induced during active infection with *Chlamydia*.

NOD1 and NOD2 can drive NF-κB-dependent inflammatory responses following the recognition of peptidoglycan products in the cytosol of an infected cell (23). Mutations in NOD2 are associated with a higher risk of developing chronic inflammatory disorders such as Crohn’s disease (28, 29), providing a link between the activity of these pathogen sensors and chronic inflammation. *Chlamydia* expresses peptidoglycan and would therefore have the ability to activate NOD-dependent pathways via established cytosolic sensing pathways (20). Indeed, chlamydial peptidoglycan has been shown to activated NOD2 (20), and NOD1 is required for maximal type I interferon (IFN) by HeLa cells after *Chlamydia* infection *in vitro* (18, 21). Other studies show that *in vitro* knockdown of NOD1 or RIP2 can substantially reduce the production of IL-8 *in vitro* (30). Our *in vivo* infection data largely agree with these previous reports and demonstrate that NOD1/2- and RIP2-deficient mice have a significantly reduced ability to control *Chlamydia* infection. Previous studies examining single NOD1- and RIP2-deficient mice have failed to detect a significant effect of these molecules on *Chlamydia* shedding. While these previous results are somewhat discordant with our present data, it should be noted that the enhanced shedding of bacteria was modest in double NOD1/2-deficient mice and only detectable during the early stage of infection. While the effect of RIP2 deficiency was more substantial in our study, this difference was probably amplified by our use of a systemic infection to monitor cytokine production and bacterial growth in an easily accessible tissue. Together, these data suggest a redundant role for NOD1/2 and RIP2 signaling in the development of inflammatory responses to *Chlamydia* infection.

Of perhaps greater interest is our finding that the UPR pathway is a critical component of the inflammatory response to *Chlamydia* infection. Our previous experiments showed that an IRE1α kinase inhibitor or a dominant negative form of RIP2 blunted IL-6 production induced during C. muridarum infection of HeLa cells (26). This *in vitro* data suggested that NOD1/NOD2/RIP2 signaling might play a critical role in linking the ER stress response to the induction of inflammation. Our present data show that TUDCA treatment can effectively block cytokine production and bacterial clearance *in vivo*, suggesting that the ER stress response represents a key component of host responses to *Chlamydia* infection *in vivo*. These data provide an alternative explanation for the role of NOD pathways in *Chlamydia*-induced inflammatory responses. Although *Chlamydia* can produce peptidoglycan peptides that induce NOD activation, it is not clear that this is how these receptors sense infection of host cells. This alternative pathway of NOD activation allows host cells to respond more generally to pathogens that induce ER stress in the absence of a requirement for peptidoglycan sensing. However, it should be noted that our use of IL-6 measurements to identify the ER stress inflammation does not necessarily mean that IL-6 plays a direct role in this process. Indeed, IL-6 could serve as useful marker of ER stress, but other cytokines play a more prominent role in bacterial clearance or driving the inflammatory response. Future studies blocking IL-6 or using IL-6-deficient mice will be required to test this rigorously.

Given the known role of *Chlamydia* in co-opting the ER for lipid and nutrient acquisition, it seems likely that this pathway is operational during active infection of the reproductive tract. Indeed, we would speculate that the IL-6 response detected in the spleen is reflective of the natural response to bacterial infection at other tissue sites, including the reproductive tract. However, analysis of the ER stress response within the reproductive tract is likely to be challenging, since it represents one of many potential pathways for induction of *Chlamydia* immunity. Therefore, our focus in this initial study was the splenic response to intravenous infection. Examination of the reproductive tract in more detail will require better definition of the cell types harboring bacteria in the submucosa and better visualization tools for analysis of cell type-specific responses. Another limitation of our study is the analysis of ER stress on inflammation as it pertains to bacterial burdens rather than reproductive tract pathology. This outcome will be important to incorporate into future assessments of ER stress responses in the reproductive tract.

Together, our data add further support to a role for NOD in the generation of inflammatory responses that lead to bacterial clearance during *Chlamydia* infection. These data also show a link between ER stress and the induction of NOD pathways, providing a ligand-independent pathway that may lead to host inflammation during infection. Greater understanding of these detailed mechanisms may provide a framework for understanding host resistance to *Chlamydia* and the development of reproductive tract pathology.

## MATERIALS AND METHODS

### Mice.

C57BL/6 and B6.129S1-*Ripk2^tm1Flv^*/J mice were purchased from The Jackson Laboratory (Bar Harbor, ME) at 6 to 8 weeks of age. NOD1/NOD2 mice were bred in-house. All mice used for experiments were 8 to 16 weeks old, unless specifically noted. Mice were maintained under specific-pathogen-free (SPF) conditions, and all mouse experiments were performed in accordance with University of California Davis Research Animal Resource guidelines.

### Bacterial strains.

Chlamydia muridarum was purchased from ATCC and cultured in HeLa 229 cells (also obtained from ATCC) in Dulbecco’s modified Eagle’s medium supplemented with 10% fetal bovine serum. Elementary bodies (EBs) were purified by discontinuous density gradient centrifugation as previously described and stored at −80°C (31). Purified EBs were titrated by infection of HeLa 229 cells, and inclusions stained with an anti-*Chlamydia* MOMP antibody (Mo33b) (32), kindly provided by the Caldwell laboratory, were enumerated. A fresh aliquot was thawed and used for every infection experiment.

### *Chlamydia* infection and enumeration.

For systemic infection, mice were injected intravenously in the lateral tail vein with 2 × 10^5^ C. muridarum. To enumerate the bacterial burden, spleens were homogenized using glass beads to disrupt cells. After shaking for 5 min and centrifuging at 500 × *g* for 10 min, supernatants were collected and serial dilutions were plated on HeLa 229 cells. The number of inclusion-forming units (IFUs) was determined by *in vitro* infection of HeLa 229 cells, and inclusions stained with immune serum extracted from C. muridarum-infected mice were enumerated. For intravaginal infection, mice were synchronized by subcutaneous injection of 2.5 mg medroxyprogesterone (Depo-Provera; Greenstone, NJ), 7 days prior to intravaginal infection. For infection, 1 × 10^5^ C. muridarum in 5 μl sucrose-phosphate-glutamic acid (SPG) buffer was deposited in the vaginal vault. To enumerate bacteria, vaginal swabs were collected and shaken with glass beads, and serial dilutions were plated on HeLa 229 cells.

### Chemical injection.

Tauroursodeoxycholate (TUDCA; Sigma-Aldrich) was resuspended in water at 100 mg/ml and stored at −80°C. Mice were injected intraperitoneally (i.p.) daily with TUDCA following *Chlamydia* infection at the effective dose of 250 mg/kg.

### IL-6 ELISA.

Infected and uninfected mice were euthanized, and blood was collected from the thoracic cavity and placed on ice to allow clotting. Samples were centrifuged, and serum was stored at −20°C. Enzyme-linked immunosorbent assay (ELISA) kits for murine IL-6 (Ready-Set-Go kit; eBioscience) were used according to the manufacturer’s instructions, and serum cytokine concentrations were determined using standard curves of the protein standard provided.

### RNA extraction and real-time quantitative PCR.

Splenic tissues were collected, stabilized in RNAlater (Ambion), and stored at −80°C until processing. Total RNA was isolated using an RNeasy Plus Universal minikit (Qiagen Inc.). Approximately 10 mg of tissue was disrupted twice in a 2-ml tube containing 600 μl RLT lysis buffer (RNeasy Plus minikit) and 1.4 mm Matrix D ceramic beads (MP Biomedicals) by a 2010 Geno/Grinder instrument (SPEX SamplePrep) at 1,750 rpm for 3 min with 1-min rests on ice between the two runs. After centrifugation, the supernatant was collected and stored at −80°C until it was processed. First-strand cDNA was generated by using random hexamers and MultiScribe reverse transcriptase (TaqMan reverse transcription reagents) and then stored at −20°C. For quantification of *il-6*, *chop*, and *hsp5α* mRNA expression, real-time quantitative PCR was performed using a 7900HT Fast real-time PCR system (Applied Biosystems). Each reaction mixture contained 25 ng cDNA template, Fast SYBR green master mix (Applied Biosystems), and 400 nM each forward and reverse primers (Invitrogen). All reactions were performed in duplicates with the following cycles: 50°C for 2 min, 95°C for 10 min, and 40 cycles of 95°C for 15 s and 60°C for 1 min. Relative gene expressions were then calculated by the threshold cycle (2^−ΔΔ^*^CT^*) method using β-actin as the reference gene and a naive group as the reference condition.

### Statistical analyses.

All statistical analyses were performed as described in the figure legends with GraphPad Prism version 7. Statistical analyses were performed by Student's *t* test. Significance testing was performed with the nonparametric Mann-Whitney test.
